# Supplementary material for: Systemic Oncological Treatments versus Supportive Care for Patients with Advanced Hepatobiliary Cancers: An Overview of Systematic Reviews
Source: Cancers (Basel). 2023 Jan 26;15(3):766. doi: 10.3390/cancers15030766 (PMC9913533; doi:10.3390/cancers15030766)
Supplement: Supplementary file 1 [file cancers-15-00766-s001.zip › cancers-2089560-supplementary.pdf]

Supplementary materials

Table S1. Initial search strategy for MEDLINE/PubMed.

Search strategy detailed in this appendix was common for all the ASTAC project overviews, and was conducted from inception until December 2019.

#1	("Gastrointestinal Neoplasms"[Mesh:NoExp] OR "Esophageal Neoplasms"[Mesh] OR "Stomach Neoplasms"[Mesh])
#2	((esophag*[Title] OR oesophag*[Title] OR stomach*[Title] OR gastric*[Title] OR gastroesophag*[Title] OR gastrointestinal*[Title]) AND (cancer*[Title] OR carcinom*[Title] OR neoplasm*[Title] OR tumor*[Title] OR tumour*[Title] OR malignan*[Title] OR adenocar*[Title] OR oncolog*[Title]))
#3	(#1 OR #2)
#4	("Palliative Care"[Mesh] OR "Terminal Care"[Mesh] OR "Neoplasm Metastasis"[Mesh])
#5	(palliative*[Title/Abstract] OR end of life*[Title/Abstract] OR end of live*[Title/Abstract] OR terminal*[Title/Abstract] OR metasta*[Title/Abstract] OR BSC[Title/Abstract] OR supportive care*[Title/Abstract] OR advanced*[Title/Abstract] OR unresect*[Title/Abstract] OR irresect*[Title/Abstract] OR nonresect*[Title/Abstract] OR non resect*[Title/Abstract] OR inopera*[Title/Abstract] OR unopera*[Title/Abstract] OR nonopera*[Title/Abstract] OR non opera*[Title/Abstract] OR non-opera*[Title/Abstract] OR stage IV[Title/Abstract])
#6	(#4 OR #5)
#7	(#3 AND #6)
#8	("Antineoplastic Protocols"[Mesh] OR "Chemoradiotherapy"[Mesh] OR "Induction Chemotherapy"[Mesh] OR "Maintenance Chemotherapy"[Mesh] OR "Consolidation Chemotherapy"[Mesh])
#9	(antineoplastic*[Title/Abstract] OR antineoplasic*[Title/Abstract] OR chemotherap*[Title/Abstract] OR chemoradiotherap*[Title/Abstract] OR radiochemotherap*[Title/Abstract] OR carboplatin*[Title/Abstract] OR cisplatin*[Title/Abstract] OR fluorouracil*[Title/Abstract] OR 5-FU[Title/Abstract] OR capecitabine*[Title/Abstract] OR docetaxel*[Title/Abstract] OR epirucibin*[Title/Abstract] OR irinotecan*[Title/Abstract] OR

	oxaliplatin*[Title/Abstract] OR paclitaxel*[Title/Abstract] OR trifluridine*[Title/Abstract] OR tipiracil*[Title/Abstract])
#10	(#8 OR #9)
#11	("Molecular Targeted Therapy"[Mesh] OR "Antibodies, Monoclonal"[Mesh] OR "Cancer Vaccines"[Mesh])
#12	(Target*[Title/Abstract] OR antibod*[Title/Abstract] OR immunotherap*[Title/Abstract] OR vaccine[Title/Abstract] OR vaccines[Title/Abstract] OR vaccination[Title/Abstract] OR HER2[Title/Abstract] OR HER-2[Title/Abstract] OR egfr[Title/Abstract] OR VEGF*[Title/Abstract] OR HGF[Title/Abstract] OR MET[Title/Abstract] OR claudin*[Title/Abstract] OR MMP-9[Title/Abstract][JP1] OR tyrosine kinase inhibit*[Title/Abstract] OR trastuzumab[Title/Abstract] OR bevacizumab[Title/Abstract] OR rilotumumab[Title/Abstract] OR onartuzumab[Title/Abstract] OR ramucirumab[Title/Abstract] OR cetuximab[Title/Abstract] OR panitumumab[Title/Abstract] OR nimotuzumab[Title/Abstract] OR claudiximab[Title/Abstract] OR apatinib[Title/Abstract] OR lapatinib[Title/Abstract] OR regorafenib[Title/Abstract] OR everolimus[Title/Abstract] OR nivolumab[Title/Abstract] OR pembrolizumab[Title/Abstract] OR avelumab[Title/Abstract] OR durvalumab[Title/Abstract] OR ipilimumab[Title/Abstract] OR checkpoint inhibit*[Title/Abstract])
#13	(#11 OR #12)
#14	(#10 OR #13)
#15	(#7 AND #14)
#16	systematic[sb]
#17	(#15 AND #16)
#18	(animals [mh] NOT humans [mh])
#19	(#17 NOT #18)

The detailed search strategy for all databases is available on https://osf.io/7chx6/

Table S2. Updated search strategy for MEDLINE/PubMed

Search strategy detailed in this appendix was exclusive for this overview, and comprehended from December 2019 to August 2022.

#1	"Gastrointestinal Neoplasms"[Mesh:NoExp] OR "Liver Neoplasms"[Mesh] OR "Biliary Tract Neoplasms"[Mesh]
#2	(liver*[Title] OR hepatic*[Title] OR hepatocel*[Title] OR biliary tract*[Title] OR bile duct*[Title] OR gallbladder*[Title] OR gall bladder*[Title]) AND (cancer*[Title] OR carcinom*[Title] OR neoplasm*[Title] OR tumor*[Title] OR tumour*[Title] OR malignan*[Title] OR adenocar*[Title] OR oncolog*[Title])
#3	#1 OR #2
#4	"Palliative Care"[Mesh] OR "Terminal Care"[Mesh] OR "Neoplasm Metastasis"[Mesh]
#5	palliative*[Title/Abstract] OR end of life*[Title/Abstract] OR end of live*[Title/Abstract] OR terminal*[Title/Abstract] OR metasta*[Title/Abstract] OR BSC[Title/Abstract] OR supportive care*[Title/Abstract] OR advanced*[Title/Abstract] OR unresect*[Title/Abstract] OR irresect*[Title/Abstract] OR nonresect*[Title/Abstract] OR non resect*[Title/Abstract] OR inopera*[Title/Abstract] OR unopera*[Title/Abstract] OR nonopera*[Title/Abstract] OR non opera*[Title/Abstract] OR non-opera*[Title/Abstract] OR stage IV[Title/Abstract]
#6	#4 OR #5
#7	#3 AND #6
#8	"Antineoplastic Protocols"[Mesh] OR "Chemoradiotherapy"[Mesh] OR "Induction Chemotherapy"[Mesh] OR "Maintenance Chemotherapy"[Mesh] OR "Consolidation Chemotherapy"[Mesh] OR "Molecular Targeted Therapy"[Mesh] OR "Antibodies, Monoclonal"[Mesh] OR "Cancer Vaccines"[Mesh]
#9	antineoplastic*[Title/Abstract] OR antineoplasic*[Title/Abstract] OR chemotherap*[Title/Abstract] OR chemoradiotherap*[Title/Abstract] OR radiochemotherap*[Title/Abstract] OR carboplatin*[Title/Abstract] OR

	cisplatin*[Title/Abstract] OR fluorouracil*[Title/Abstract] OR 5-FU[Title/Abstract] OR capecitabine*[Title/Abstract] OR docetaxel*[Title/Abstract] OR epirucibin*[Title/Abstract] OR irinotecan*[Title/Abstract] OR oxaliplatin*[Title/Abstract] OR paclitaxel*[Title/Abstract] OR trifluridine*[Title/Abstract] OR tipiracil*[Title/Abstract] OR Target*[Title/Abstract] OR antibod*[Title/Abstract] OR immunotherap*[Title/Abstract] OR vaccine[Title/Abstract] OR vaccines[Title/Abstract] OR vaccination[Title/Abstract] OR HER2[Title/Abstract] OR HER-2[Title/Abstract] OR egfr[Title/Abstract] OR VEGF*[Title/Abstract] OR HGF[Title/Abstract] OR MET[Title/Abstract] OR claudin*[Title/Abstract] OR MMP-9[Title/Abstract] OR tyrosine kinase inhibit*[Title/Abstract] OR trastuzumab[Title/Abstract] OR bevacizumab[Title/Abstract] OR rilotumumab[Title/Abstract] OR onartuzumab[Title/Abstract] OR ramucirumab[Title/Abstract] OR cetuximab[Title/Abstract] OR panitumumab[Title/Abstract] OR nimotuzumab[Title/Abstract] OR claudiximab[Title/Abstract] OR apatinib[Title/Abstract] OR lapatinib[Title/Abstract] OR regorafenib[Title/Abstract] OR everolimus[Title/Abstract] OR nivolumab[Title/Abstract] OR pembrolizumab[Title/Abstract] OR avelumab[Title/Abstract] OR durvalumab[Title/Abstract] OR ipilimumab[Title/Abstract] OR checkpoint inhibit*[Title/Abstract]
#10	#8 OR #9
#11	#7 AND #10
#12	systematic[sb]
#13	#11 AND #12
#14	animals [mh] NOT humans [mh]
#15	#13 NOT #14

Table S3. Excluded references after full-text screening, with reasons.

Study ID	DOI	Reason for exclusion
Abdel-Rahman 2013	10.1007/s10620-013-2872-x	Wrong comparator
Abdel-Rahman 2014	10.1016/j.critrevonc.2013.12.013	Wrong comparator
Abdel-Rahman 2014	10.1586/14737140.2015.985660	Wrong comparator
Abdel-Rahman 2015	10.1586/14737140.2015.978295	Wrong study design
Abdel-Rahman 2016	10.1002/14651858.CD011313.pub2	Wrong intervention
Abdel-Rahman 2017	10.1002/14651858.CD011314.pub2	Wrong intervention
Abdel-Rahman 2017	10.1080/17474124.2017.1264874	Wrong comparator
Abdel-Rahman 2018	10.1002/14651858.CD011746.pub2	Duplicate
Abou-Alfa 2018	10.1200/JCO.2018.36.4-suppl.TPS545	Wrong publication type
Ahmed 2004	10.1002/14651858.CD003445.pub2	Wrong publication type
Ajani 2006	10.1002/cncr.21986	Wrong study design
Al-Batran 2010	10.1002/cncr.25064	Wrong publication type
Ali Abdulnabi Mohamed	No DOI	Wrong publication type
Amdal 2013	10.3109/0284186X.2012.731521	Wrong comparator
Arshad 2013	10.1097/COC.0b013e3182124216	Wrong comparator
Aya El Helali	No DOI	Wrong publication type
Azria 2008	10.1684/bdc.2008.0749	Wrong study design
Baek 2012	10.1007/s10120-011-0114-5	Wrong comparator
Baldo 2015	10.1002/14651858.CD011463	Wrong publication type
Beller 2015	10.1002/14651858.CD010206.pub2	Wrong intervention
Bennetts 2017	10.1016/j.jval.2017.08.107	Wrong comparator
Bian 2019	10.1016/j.ijsu.2018.11.010	Wrong comparator
Brown 2006	No DOI	Wrong study design
Cabalag 2015	10.1007/s10120-014-0388-5	Wrong comparator
Cabibbo 2009	No DOI	Wrong study design
Cabibbo 2010	10.1002/hep.23485	Wrong intervention
Cao 2010	10.1097/MPA.0b013e3181bdc6b8	Wrong comparator
Carter 2015	10.3111/13696998.2015.1066380	Wrong outcomes
Casaretto 2006	10.1590/s0100-879x2006000400002	Wrong publication type
Chan 2013	No DOI	Wrong comparator

Chan 2016	No DOI	Wrong study design
Chan 2017a	10.1371/journal.pone.0172307	Wrong patient population
Chan 2017b	10.1016/j.critrevonc.2017.05.002	Wrong patient population
Chan 2019	10.1177/1758835919859990	Wrong study design
Chau 2019	10.1200/JCO.2019.37.4_suppl.128	Wrong publication type
Chen 2013	10.1371/journal.pone.0060320	Wrong comparator
Chen 2018	10.2147/OTT.S157466	Wrong study design
Chen 2019	10.1080/2162402X.2019.1581547	Wrong patient population
Chen 2019	10.3892/mmr.2018.9638	Wrong comparator
Cherny 2004	10.1093/annonc/mdh928	Wrong study design
Cherny 2009	10.1200/JCO.2009.21.9592	Wrong study design
Chin 2014	No DOI	Wrong publication type
Chin 2018	10.1002/14651858.CD011044.pub2	Wrong patient population
Chintalacheruvu 2017	No DOI	Wrong publication type
Chow 1998	0.1002/14651858.CD001403	Wrong publication type
Chow 2005	10.1016/j.ctrv.2005.07.005	Wrong intervention
Chu 2015	No DOI	Wrong study design
Chua 2006	10.1016/j.bpg.2005.10.003	Wrong study design
Cinar 2017	10.21037/cco.2017.06.13	Wrong study design
Citterio 2018	10.18632/oncotarget.25639	Wrong comparator
Cowley 2017	10.11124/JBISRIR-2016-003108	Wrong comparator
Cuyun Carter 2014	10.1016/j.jval.2014.03.536	Wrong intervention
Dean 2019	10.1200/JCO.2019.37.15_suppl.e15794	Wrong comparator
Delos Santos 2020	10.3747/co.27.6583	Wrong study design
Desiderio 2017	10.1016/j.ejca.2017.03.030	Wrong comparator
Di Giorgio 2019	10.23736/S0026-4806.19.06081-6	Wrong comparator
Dingle 2005	10.1155/2005/565479	Wrong patient population
Dong 2019	10.5246/JCPS.2019.04.027	Wrong intervention
Duffy 2013	10.1002/hep.26120	Wrong study design
Eckel 2007	10.1038/sj.bjc.6603648	Wrong comparator
Eltawil 2012	10.1111/j.1477-2574.2012.00441.x	Wrong comparator
Facchiano 2012	10.1245/s10434-012-2360-0	Wrong comparator

Fantini 2015	No DOI	Wrong publication type
Feingold 2017	10.1002/jso.24476	Wrong intervention
Fornaro 2015	10.1093/annonc/mdv233.147	Wrong intervention
Gandara-Ladron de Guevara 2014	10.1136/ejhpharm-2013-000436.208	Wrong comparator
Genglong Liu	No DOI	Wrong publication type
Gentile 2019	10.1016/S0016-5085%2819%2940738-5	Wrong intervention
Glimelius 1998	No DOI	Wrong study design
Gollala 2016	No DOI	Wrong comparator
Grassadonia 2018	10.3390/jcm7120542	Wrong patient population
Gresham 2014	10.1186/1471-2407-14-471	Wrong comparator
Hajatdoost 2018	10.3390/medicina54030048	Wrong comparator
Hall 2018	10.1245/s10434-018-6349-1	Wrong comparator
Harvey 2017	10.1007/s40487-017-0048-0	Wrong patient population
Haun 2017	10.1002/14651858.CD011129.pub2	Wrong intervention
Hill 2006	No DOI	Wrong study design
Hoeben 2016	10.1093/annonc/mdw183	Wrong comparator
Homs 2006		Duplicate
Homs 2010	10.1002/14651858.CD004063.pub3	Duplicate
Hsu 2012	10.1007/s10120-011-0106-5	Wrong comparator
Hu 2017	10.1111/jcpt.12498	Wrong comparator
Huang	No DOI	Wrong comparator
Huang 2017	10.5114/wo.2017.66653	Wrong comparator
Iacovelli 2014	10.1371/journal.pone.0108940	Wrong patient population
Janmaat 2016	10.1177/2050640616663688	Duplicate
Janmaat 2017	10.1002/14651858.CD004063.pub4	Wrong patient population
Janmaat 2017	10.1002/14651858.CD004063.pub4	Duplicate
Janowitz 2016	10.1038/bjc.2015.452	Wrong study design
Jen 2015	10.1093/annonc/mdv523.63	Wrong publication type
Jian Yang	No DOI	Wrong publication type
Jiuda Zhao	No DOI	Wrong comparator
Kasuga 2016	No DOI	Wrong publication type
Kasuga 2018	10.1007/s10637-018-0589-6	Wrong comparator

Katz 2019	10.1007/s12029-019-00243-8	Wrong study design
Kerui Wu	No DOI	Wrong comparator
Kim 2013	10.1093/annonc/mdt351	Wrong study design
Kim 2017	10.18632/oncotarget.18314	Wrong study design
Kocher 2010	No DOI	Wrong study design
Kordes 2017	No DOI	Wrong publication type
Kristensen 2016	10.1016/j.critrevonc.2016.01.006	Wrong study design
Lamarca 2014	10.1093/annonc/mdu162	Wrong comparator
Lamarca 2019	10.1093/jnci/djz071	Wrong comparator
Lee 2015	10.1093/annonc/mdv207	Wrong outcomes
Lee 2018	10.1136/bmjopen-2017-017249	Wrong publication type
Lei 2017	10.1097/MD.0000000000006301	Other reasons
Li 2019	10.3389/fonc.2019.00441	Wrong study design
Li 2019	10.1097/MD.0000000000016108	Wrong intervention
Lim 2021	10.1093/jnci/djaa119	Wrong study design
Liu 2010	10.1007/s00280-009-1090-x	Wrong publication type
Liu 2014	10.1371/journal.pone.0091124	Wrong comparator
Liu 2016	10.1097/MD.0000000000004993	Wrong comparator
Liu 2018	10.18632/oncotarget.23429	Wrong patient population
Liu 2019	10.1016/j.jcyt.2019.07.006	Wrong patient population
Llovet 2003	10.1053/jhep.2003.50047	Wrong comparator
Mattiucci 2014	10.1016/j.critrevonc.2013.10.007	Wrong intervention
McNamara 2018	10.1016/j.ejca.2018.09.031	Wrong comparator
Montero 2005	10.1016/S1470-2045%2805%2970094-2	Wrong comparator
Moole 2016	No DOI	Wrong intervention
Moriwaki 2016	10.1038/bjc.2016.83	Wrong study design
Nagrial 2013		Wrong publication type
Niu 2016	10.1097/MD.0000000000005591	Wrong study design
Nowak 2004	10.1002/14651858.CD001024.pub2	Wrong intervention
Permert 2001	10.1080/02841860151116448	Wrong study design
Qi 2013	10.1002/ijc.27775	Wrong comparator
Qi 2016	10.18632/oncotarget.12102	Wrong study design

Rahma 2013	10.1093/annonc/mdt166	Wrong study design
Roccarina 2017	10.1002/14651858.CD011649.pub2	Wrong patient population
Roccarina 2017	10.1002/14651858.CD011649.pub2	Duplicate
Rossi 2015	10.1002/14651858.CD011568	Wrong publication type
Roviello	No DOI	Wrong publication type
Scartozzi 2007	10.1517/14656566.8.6.797	Wrong study design
Shan 2014	10.1111/ajco.12305	Duplicate
Sharma 2011	10.3748/wjg.v17.i7.867	Wrong study design
Shen 2013	10.1097/MCG.0b013e3182a87cfd	Wrong patient population
Shi 2017	10.1038/s41598-017-05464-0	Wrong comparator
Simonetti 1997	10.1023/a:1008285123736	Wrong study design
Sreedharan 2009	10.1002/14651858.CD005048.pub2	Wrong intervention
Suker 2016	10.1016/S1470-2045(16)00172-8	Wrong comparator
Sultana 2007	10.1038/sj.bjc.6603719	Wrong study design
Sultana 2007	10.1200/JCO.2006.09.2551	Wrong publication type
Sultana 2008	10.1038/sj.bjc.6604436	Wrong comparator
Sultana 2014	10.1002/14651858.CD011044	Wrong patient population
Sun 2013	10.1186/1471-2407-13-577	Wrong intervention
Sun 2017	10.18632/oncotarget.20445	Wrong study design
Tang 2018	10.1097/MD.0000000000013525	Wrong intervention
Tassinari 2015	10.1093/annonc/mdv344.23	Wrong publication type
Tassinari 2015	No DOI	Duplicate
Ter Veer 2016	10.1007/s10120-015-0587-8	Wrong comparator
TerVeer 2016	10.1007/s10555-016-9632-2	Wrong patient population
TerVeer 2018	10.1007/s10120-018-0792-3	Wrong outcomes
The GASTRIC Group 2013	10.1016/j.ejca.2012.12.016	Wrong study design
Tomita 2016	10.1002/14651858.CD012078	Wrong publication type
Tremblay 2017	10.1093/annonc/mdx369.091	Wrong publication type
Tremblay 2017	No DOI	Duplicate
Tremblay 2017	10.1016/j.jval.2017.08.096	Duplicate
vanKleef 2019	10.1007/s11136-018-1946-9	Wrong patient population
Victor H. F. de Jesus 2020	No DOI	Wrong publication type

Wagner 2005	10.1002/14651858.CD004064.pub2	Duplicate
Wagner 2006	10.1200/JCO.2005.05.0245	Duplicate
Wagner 2010	10.1002/14651858.CD004064.pub3	Duplicate
Wagner 2017	10.1002/14651858.CD004064.pub4	Wrong patient population
Wagner 2017	10.1002/14651858.CD004064.pub4	Duplicate
Wallis 2019	10.1001/jamaoncol.2018.5904	Wrong patient population
Walma 2016	No DOI	Wrong intervention
Wang 2016	PMID: 27212163	Wrong patient population
Wang 2016	10.2147/DDDT.S105442	Wrong comparator
Wang 2017	10.2147/OTT.S110431	Wrong patient population
Wang 2017	10.1002/cam4.1156	Wrong comparator
Wang 2018	10.1097/MD.0000000000010164	Wrong comparator
Wang 2021	10.1016/j.clinre.2020.04.010	Wrong intervention
Weis 2013	10.1002/14651858.CD003046.pub3	Wrong intervention
Whistance 2011	10.1097/SPC.0b013e3283436ecb	Wrong patient population
Xie 2017	10.18632/oncotarget.15923	Wrong patient population
Yang 2013	10.1371/journal.pone.0057528	Wrong comparator
Yang 2015	10.3978/j.issn.2078-6891.2015.055	Wrong intervention
Yerasi 2016	No DOI	Wrong outcomes
Yip 2006	10.1002/14651858.CD002093.pub2	Wrong patient population
Zagouri 2013	10.1097/MPA.0b013e31827aedef	Wrong comparator
Zhang 2010	10.1097/CAD.0b013e3283350e26	Wrong study design
Zhang 2018	10.1093/annonc/mdy282.111	Wrong comparator
Zhao 2018	10.1007/s10120-018-0813-2	Wrong patient population
Zheng 2017	10.1097/MD.0000000000006884	Wrong study design
Zhu 2015	10.1371/journal.pone.0128616	Wrong comparator
Zhu 2017	10.1007/s10120-016-0656-7	Wrong patient population
Zhuang 2013	10.1371/journal.pone.0061361	Wrong comparator
Ziyu Li	No DOI	Wrong publication type

Figure S1. General characteristics and risk of bias of relevant primary studies (as reported by SR authors).

Author Year	n	Patients	Type of intervention		Risk of bias*					
			Drug	First line?	Random sequence	Allocation concealment	Blinding of participants and	Blinding of outcome	Incomplete outcome data	Selective reporting
Llovet 2008 (SHARP) [1]	602	Advanced HCC	Sorafenib	Yes				?		
Cheng 2009 [2]	226	Advanced HCC	Sorafenib	Yes						
Sharma 2010 [3]	81	Unresectable GB cancer	Fluorouracil/folinic acid Gemcitabine/oxaliplatin	NS						
Hsu 2012 [4]	67	Advanced HCC	Vandetanib	Yes						
Llovet 2013 (BRISK-PS) [5]	395	Advanced HCC	Brivanib	No						?
Santoro 2013 [6]	107	Advanced HCC	Tivantinib	No						?
Rimassa 2013 [7]	101	Advanced HCC	Sorafenib	No**						?
Yen 2014 [8]	185	Advanced HCC	Codrituzumab	No						
Zhu 2014 (EVOLVE-1) [9]	546	Advanced HCC	Everolimus	No			?	?		
Ji 2014 [10]	189	Advanced HCC	Sorafenib	Yes						
Zhu 2015 (REACH) [11]	565	Advanced HCC	Ramucirumab	No			?	?		
Kang 2015 [12]	202	Advanced HCC	Axitinib	No				?		?

Abou-Alfa 2016 [13]	185	Advanced HCC	Codrituzumab	No						?
Bruix 2017 (RESORCE) [14]	573	Advanced HCC	Regorafenib	No						
Kudo 2017 (S-CUBE) [15]	334	Advanced HCC	S-1	No				?		?
Rimassa 2018 (METIV-HCC) [16]	340	Advanced HCC	Tivantinib	No						
Abou-Alfa 2018 [17]	635	Advanced HCC	ADI-PEG20	No						?
Abou-Alfa 2018b (CELESTIAL) [18]	707	Advanced HCC (increased AFP)	Cabozantinib	No						?
Zhu 2019 (REACH-2) [19]	292	Advanced HCC	Ramucirumab	No			?	?		
Finn 2019 (KEYNOTE-240) [20]	413	Advanced HCC	Pembrolizumab	No			?			
Li 2020*** [21]	393	Advanced HCC	Apatinib	No						
Kudo 2020 (JET-HCC) [22]	195	Advanced HCC	Tivantinib	No						

AFP: alpha-fetoprotein; GB: gallbladder.

*Green cells represent low risk of bias. Yellow cells represent unclear risk of bias. Red cells represent high risk of bias. This section summarises the majority of assessments made by SR authors

**Dose escalation versus BSC

***Assessment not disaggregated for this study

?: Represents unclear assessments by overview authors, due to simultaneous low and high risk assessments made by two or more different SRs

NS: Not specified

References

1. Llovet JM, Ricci S, Mazzaferro V, Hilgard P, Gane E, Blanc JF, et al. Sorafenib in advanced hepatocellular carcinoma. N Engl J Med. 2008 Jul 24;359(4):378–90.

2. Cheng AL, Kang YK, Chen Z, Tsao CJ, Qin S, Kim JS, et al. Efficacy and safety of sorafenib in patients in the Asia-Pacific region with advanced hepatocellular carcinoma: a phase III randomised, double-blind, placebo-controlled trial. Lancet Oncol. 2009 Jan;10(1):25–34.

3. Sharma A, Dwary AD, Mohanti BK, Deo SV, Pal S, Sreenivas V, et al. Best supportive care compared with chemotherapy for unresectable gall bladder cancer: a randomized controlled study. J Clin Oncol. 2010 Oct 20;28(30):4581–6.

4. Hsu C, Yang TS, Huo TI, Hsieh RK, Yu CW, Hwang WS, et al. Vandetanib in patients with inoperable hepatocellular carcinoma: a phase II, randomized, double-blind, placebo-controlled study. J Hepatol. 2012 May;56(5):1097–103.

5. Llovet JM, Decaens T, Raoul JL, Boucher E, Kudo M, Chang C, et al. Brivanib in patients with advanced hepatocellular carcinoma who were intolerant to sorafenib or for whom sorafenib failed: results from the randomized phase III BRISK-PS study. J Clin Oncol. 2013 Oct 1;31(28):3509–16.

6. Santoro A, Rimassa L, Borbath I, Daniele B, Salvagni S, Van Laethem JL, et al. Tivantinib for second-line treatment of advanced hepatocellular carcinoma: a randomised, placebo-controlled phase 2 study. Lancet Oncol. 2013 Jan;14(1):55–63.

7. Rimassa L, Pressiani T, Boni C, Carnaghi C, Rota Caremoli E, Fagiuoli S, et al. A phase II randomized dose escalation trial of sorafenib in patients with advanced hepatocellular carcinoma. Oncologist. 2013 Apr 11;18(4):379–80.

8. Yen CJ, Daniele B, Kudo M, Merle P, Park JW, Ross PJ, et al. Randomized phase II trial of intravenous RO5137382/GC33 at 1600 mg every other week and placebo in previously treated patients with unresectable advanced hepatocellular carcinoma (HCC; NCT01507168). J Clin Oncol. 2014 May 20;32(15_suppl):4102–4102.

9. Zhu AX, Kudo M, Assenat E, Cattan S, Kang YK, Lim HY, et al. Effect of everolimus on survival in advanced hepatocellular carcinoma after failure of sorafenib: the EVOLVE-1 randomized clinical trial. JAMA. 2014 Jul 2;312(1):57–67.

10. Ji YX, Zhang ZF, Lan KT, Nie KK, Geng CX, Liu SC, et al. Sorafenib in liver function impaired advanced hepatocellular carcinoma. Chin Med Sci J. 2014 Mar;29(1):7–14.

11. Zhu AX, Park JO, Ryoo BY, Yen CJ, Poon R, Pastorelli D, et al. Ramucirumab versus placebo as second-line treatment in patients with advanced hepatocellular carcinoma following first-line therapy with sorafenib (REACH): a randomised, double-blind, multicentre, phase 3 trial. Lancet Oncol. 2015 Jul;16(7):859–70.

12. Kang YK, Yau T, Park JW, Lim HY, Lee TY, Obi S, et al. Randomized phase II study of axitinib versus placebo plus best supportive care in second-line treatment of advanced hepatocellular carcinoma. Ann Oncol. 2015 Dec;26(12):2457–63.

13. Abou-Alfa GK, Puig O, Daniele B, Kudo M, Merle P, Park JW, et al. Randomized phase II placebo controlled study of codrituzumab in previously treated patients with advanced hepatocellular carcinoma. J Hepatol. 2016 Aug;65(2):289–95.

14. Bruix J, Qin S, Merle P, Granito A, Huang YH, Bodoky G, et al. Regorafenib for patients with hepatocellular carcinoma who progressed on sorafenib treatment (RESORCE): a randomised, double-blind, placebo-controlled, phase 3 trial. Lancet. 2017 Jan 7;389(10064):56–66.

15. Kudo M, Moriguchi M, Numata K, Hidaka H, Tanaka H, Ikeda M, et al. S-1 versus placebo in patients with sorafenib-refractory advanced hepatocellular carcinoma (S-CUBE): a randomised, double-blind, multicentre, phase 3 trial. Lancet Gastroenterol Hepatol. 2017 Jun;2(6):407–17.

16. Rimassa L, Assenat E, Peck-Radosavljevic M, Pracht M, Zagonel V, Mathurin P, et al. Tivantinib for second-line treatment of MET-high, advanced hepatocellular carcinoma (METIV-HCC): a final analysis of a phase 3, randomised, placebo-controlled study. Lancet Oncol. 2018 May;19(5):682–93.

17. Abou-Alfa GK, Qin S, Ryoo BY, Lu SN, Yen CJ, Feng YH, et al. Phase III randomized study of second line ADI-PEG 20 plus best supportive care versus placebo plus best supportive care in patients with advanced hepatocellular carcinoma. Ann Oncol. 2018 Jun 1;29(6):1402–8.

18. Abou-Alfa GK, Meyer T, Cheng AL, El-Khoueiry AB, Rimassa L, Ryoo BY, et al. Cabozantinib in Patients with Advanced and Progressing Hepatocellular Carcinoma. N Engl J Med. 2018 Jul 5;379(1):54–63.

19. Zhu AX, Kang YK, Yen CJ, Finn RS, Galle PR, Llovet JM, et al. Ramucirumab after sorafenib in patients with advanced hepatocellular carcinoma and increased α-fetoprotein concentrations (REACH-2): a randomised, double-blind, placebo-controlled, phase 3 trial. Lancet Oncol. 2019 Feb;20(2):282–96.

20. Finn RS, Ryoo BY, Merle P, Kudo M, Bouattour M, Lim HY, et al. Pembrolizumab As Second-Line Therapy in Patients With Advanced Hepatocellular Carcinoma in KEYNOTE-240: A Randomized, Double-Blind, Phase III Trial. J Clin Oncol. 2020 Jan 20;38(3):193–202.

21. Li Q, Qin S, Gu S, Chen X, Lin L, Wang Z, et al. Apatinib as second-line therapy in Chinese patients with advanced hepatocellular carcinoma: A randomized, placebo-controlled, double-blind, phase III study. J Clin Oncol. 2020 May 20;38(15_suppl):4507–4507.

22. Kudo M, Morimoto M, Moriguchi M, Izumi N, Takayama T, Yoshiji H, et al. A randomized, double-blind, placebo-controlled, phase 3 study of tivantinib in Japanese patients with MET-high hepatocellular carcinoma. Cancer Sci. 2020 Oct;111(10):3759–69.

Figure S2. Summary of Findings tables.

Sorafenib compared to placebo/UPSC for patients with advanced HCC as first line therapy

Patient or population: patients with advanced HCC as first line therapy
Setting:
Intervention: sorafenib
Comparison: placebo/UPSC

Outcomes	№ of participants (studies) Follow-up	Certainty of the evidence (GRADE)	Relative effect (95% CI)	Anticipated absolute effects	
				Risk with placebo/UPSC	Risk difference with sorafenib
Overall survival (OS)	1017 (3 RCTs)	⊕⊕⊕⊕ High[a]	**HR 0.62** (0.50 to 0.77) [Overall survival]	**1 year risk** 843 per 1.000	**160 fewer per 1.000** (239 fewer to 83 fewer)
Quality of life - not reported	-	-			
Functional status - not reported	-	-			
Toxicity (AEs) assessed with: Adverse events grade 3 or more	823 (2 RCTs)	⊕◯◯◯ Very low[b]	**RR 1.18** (0.87 to 1.60)	175 per 1.000	**32 more per 1.000** (23 fewer to 105 more)
Progression-free survival - not reported	-	-	-	-	-

*__The risk in the intervention group__ (and its 95% confidence interval) is based on the assumed risk in the comparison group and the **relative effect** of the intervention (and its 95% CI).

CI: confidence interval; **HR:** hazard Ratio; **RR:** risk ratio

GRADE Working Group grades of evidence
High certainty: we are very confident that the true effect lies close to that of the estimate of the effect.
Moderate certainty: we are moderately confident in the effect estimate: the true effect is likely to be close to the estimate of the effect, but there is a possibility that it is substantially different.
Low certainty: our confidence in the effect estimate is limited: the true effect may be substantially different from the estimate of the effect.
Very low certainty: we have very little confidence in the effect estimate: the true effect is likely to be substantially different from the estimate of effect.

Explanations

a. Ji 2014 had unclear risk of selection and selective reporting bias, and Llovet 2008 (SHARP) had unclear risk of detection bias due to inconsistent assessment of SR authors. However, we decided not do downgrade certainty of evidence for this domain, because the unclear risk of bias of Ji 2014 is probably due to its publication status (abstract), and Llovet 2008 (SHARP) was mostly assessed as low risk of bias by SR authors.
b. Downgraded three levels of certainty of evidence due to imprecision. We considered 5%, 10% and 15% as thresholds to consider the change in the events as small, moderate or large, respectively. In this case, the wide conficence intervals cross these thresholds, and number of events/participants do not meet the optimal information size

Systemic oncological treatments compared to placebo/UPSC for patients with advanced HCC as second line therapy

Patient or population: patients with advanced HCC as second line therapy
Setting:
Intervention: systemic oncological treatments
Comparison: placebo/UPSC

Outcomes	№ of participants (studies) Follow-up	Certainty of the evidence (GRADE)	Relative effect (95% CI)	Anticipated absolute effects	
				Risk with placebo/UPSC	Risk difference with systemic oncological treatments
Overall survival (OS)	6168 (17 RCTs)	⊕⊕⊕◯ Moderate[a,b,c]	HR 0.85 (0.79 to 0.92) [Overall survival]	1 year risk	
				843 per 1.000	50 fewer per 1.000 (75 fewer to 25 fewer)
Quality of life (QoL)	(1 RCT)	⊕⊕◯◯ Low[d]	Quality of life was similar for the comparison regorafenib versus placebo/BSC [e]		
Functional status - not reported	-	-			
Toxicity (AEs) assessed with: Adverse events grade 3 or more	2964 (6 RCTs)[f]	⊕⊕◯◯ Low[g,h]	RR 1.58 (1.28 to 1.96)	416 per 1.000	242 more per 1.000 (117 more to 400 more)
Progression-free survival (PFS)	5126 (14 RCTs)	⊕⊕◯◯ Low[a,c,i]	HR 0.67 (0.55 to 0.80) [Progression-free survival]	1 year risk	
				843 per 1.000	132 fewer per 1.000 (204 fewer to 70 fewer)

***The risk in the intervention group** (and its 95% confidence interval) is based on the assumed risk in the comparison group and the **relative effect** of the intervention (and its 95% CI).

CI: confidence interval; **HR:** hazard Ratio; **RR:** risk ratio

GRADE Working Group grades of evidence
High certainty: we are very confident that the true effect lies close to that of the estimate of the effect.
Moderate certainty: we are moderately confident in the effect estimate: the true effect is likely to be close to the estimate of the effect, but there is a possibility that it is substantially different.
Low certainty: our confidence in the effect estimate is limited: the true effect may be substantially different from the estimate of the effect.
Very low certainty: we have very little confidence in the effect estimate: the true effect is likely to be substantially different from the estimate of effect.

Explanations

a. Downgraded by one level due to risk of bias, especially unclear risk of selection, performance and detection bias in many of the included studies.
b. We defined thresholds for small, moderate and large effects as 5%, 10% and 15% reduction in survival. The confidence interval crossess this thresholds, nevertheless, we did not downgrade certainty of evidence because most studies show a favourable effect, and upper bound of the confidence interval shows, at least, a favourable small effect.
c. No publication bias detected by inspection of the funnel plot.
d. Downgraded by two levels due to indirectness. The finding comes from one trial included in one systematic review, assessing only regorafenib. It is unclear whether this finding could be applicable to other SOTs. If the finding was interpreted especifically for regorafenib, then we would no downgrade certainty of evidence for this reason.
e. Only narrative information provided.
f. We included only studies that especifically reported adverse events grade 3 or higher.
g. Downgraded by one level due to risk of bias, especiallyunclear risk of selection bias in BRISK-PS, unclear risk of performance or detection bias in EVOLVE-1 and KEYNOTE-240, and overall unclear risk of reporting biases.
h. Downgrade by one level due to inconsistency, with I2=86%. Nevertheless, all the studies tend to favour placebo/UPSC.
i. Downgraded by one level due to inconsistency, with I2=86%. We performed an exploratory sensitivity analysis, removing the study by Abou Alfa 2018 (ADI-PEG20), but inconsistency remained high (I2=77%).

Chemotherapy compared to placebo/UPSC for patients with advanced gallbladder cancer					
**Patient or population:** patients with advanced gallbladder cancer<br>**Setting:**<br>**Intervention:** chemotherapy<br>**Comparison:** placebo/UPSC					
**Outcomes**	**№ of participants (studies) Follow-up**	**Certainty of the evidence (GRADE)**	**Relative effect (95% CI)**	**Anticipated absolute effects**	
				**Risk with placebo/UPSC**	**Risk difference with chemotherapy**
				**1 year risk**	
Overall survival - 1 year (OS-1yr)	81<br>(1 RCT)	⨁◯◯◯<br>Very low[a,b]	**RR 0.89**<br>(0.74 to 1.07)	843 per 1.000	**93 fewer per 1.000**<br>(219 fewer to 59 more)
Quality of life - not reported	-	-			
Functional status - not reported	-	-			
Toxicity - not reported	-	-	-	-	-
Progression-free survival - not reported	-	-	-	-	-

*__The risk in the intervention group__ (and its 95% confidence interval) is based on the assumed risk in the comparison group and the **relative effect** of the intervention (and its 95% CI).

CI: confidence interval; **RR:** risk ratio

GRADE Working Group grades of evidence
High certainty: we are very confident that the true effect lies close to that of the estimate of the effect.
Moderate certainty: we are moderately confident in the effect estimate: the true effect is likely to be close to the estimate of the effect, but there is a possibility that it is substantially different.
Low certainty: our confidence in the effect estimate is limited: the true effect may be substantially different from the estimate of the effect.
Very low certainty: we have very little confidence in the effect estimate: the true effect is likely to be substantially different from the estimate of effect.

Explanations

a. Downgraded two levels of certainty due to unclear risk of selection bias and high risk of performance bias.
b. Downgraded three levels of certainty of evidence due to imprecision. We considered 5%, 10% and 15% as thresholds to consider the change in the events as small, moderate or large, respectively. In this case, the wide conficence intervals cross these thresholds, and number of events/participants do not meet the optimal information size

Figure S3. Funnel plot for targeted/biological therapies versus placebo/ UPSC as second line therapy for advanced HCC; (a) Outcome: Overall survival, time-to-event; (b) Outcome: Progression free survival time-to-event

(a) (b)

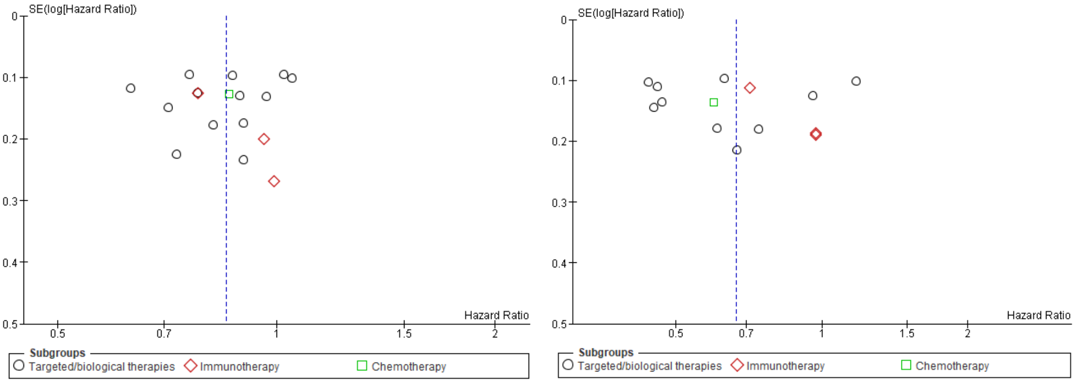

Only for outcomes with 10 or more included studies providing data for that specific comparison.
